# A systematic review of estimation of growth curve in goats

**DOI:** 10.1007/s11250-023-03857-0

**Published:** 2023-12-18

**Authors:** Ledimo Faith Makgopa, Madumetja Cyril Mathapo, Thobela Louis Tyasi

**Affiliations:** Department of Agricultural Economics and Animal Production, University of Limpopo, Private Bag X1106, Sovenga, Limpopo 0727 South Africa

**Keywords:** Brody, Richards, Gompertz, Von Bertalanffy, Logistic models

## Abstract

Growth is an economically important trait in animal production industry and is one of the subjects that can be justified mathematically. The literature recommends different non-linear model to estimate the growth of goats. The objective of this study was to systematically review the literature published on estimation of growth using non-linear models in goats. Databases such as Google Scholar, PubMed, ScienceDirect, and Web of Science were evaluated systematically using the combination of the following key terms: Non-linear growth curve models such as Brody, Richards, Gompertz, Von Bertalanffy, Logistic models. A total of 25 eligible articles were found published between 2008 and 2022 in Bangladesh, Brazil, China, Egypt, Germany, India, Indonesia, Iran, Pakistan, South Africa, Turkey, Tunisia, and Vietnam. The results showed that out of 25 articles, Gompertz growth curve model was the most used (*n* = 10), followed by Logistic (*n* = 8), then Brody growth curve model (*n* = 6). The findings further indicated that Janoscheck growth curve model was the least used model (*n* = 1) for estimation of growth in goats. One of the limitations is that some of the reviewed articles did not indicate the sex of the animals which make it difficult to draw the conclude for sexes. The systematic review concludes that Gompertz growth curve model is the most recommended for estimation of growth parameters of goats, followed by Logistic, and then Brody. Therefore, researchers should consider using these models when studying growth parameters of goats.

## Introduction

Growth is an economically important trait in animal production industry and is one of the subjects that can be justified mathematically (Waheed et al. 2011). The economic success of a small ruminant production system is influenced by the animal’s fast growth rate which dictate their meat producing potential up to marketing age (Kheirabadi and Rashidi 2019). However, growth parameters of goat are affected by several genetic and non-genetic factors at different age (Gautam et al. 2019). The growth curve parameters of goats can be predicted using non-linear models (Magotra et al. 2021). Brody, Gompertz, Von Bertalanffy, Richards, and Logistic are some of the non-linear models used to estimate biological parameters (Arré et al. 2019). Non-linear models are more preferred than linear models because the growth of an animal has a sigmoidal shape which make them suitable to describe the growth curve of goats (Rashad et al. 2022). Studies have been conducted to distinguish the growth pattern of small ruminants and models that predict weight and age data of animals (Cak et al. 2017; Waiz et al. 2019; de Sousa et al. 2021). However, to the best on our knowledge, there is no comprehensive systematic review on estimation of growth in goats using non-linear growth curve models. Therefore, this study will assist to indicate the best fit non-linear model that can be used to estimate the growth of goats. Hence, the objective of this study was to systematically review the literature published on estimation of growth in goats. The systematically reviewed outcome will provide information that will assist researchers for estimation of growth curve parameters to help goat farmers to implement goat management practices and increase their profit potential.

## Materials and methods

### Eligibility criteria

Identification of Population, Exposure, and Outcomes (PEO) components of the research question were performed for this systematic review. The “goats” were defined as the population of the study, with the “non-linear growth curve models” as exposure and “recommended non-linear models for estimation of growth curve parameters of goats” as the outcomes. Prior decided to conduct the study, an initial search of the PEO elements on Google Scholar, ScienceDirect, PubMed, and Web of Science was conducted.

### Search strategy

Two investigators (Ledimo Faith Makgopa and Thobela Louis Tyasi) performed a systematic review of articles in the databases such as Google Scholar, PubMed, ScienceDirect and Web of Science, combination of the following key terms: Brody, Richards, Gompertz, Von Bertalanffy, Logistic models. The key terms were combined in various combinations. Only English studies were considered in the study.

### Inclusion criteria

Articles that were present in more than one database were removed before screening for eligibility. The inclusion criteria were articles that evaluated growth patterns of goats using growth curve models, articles that are published in English, and articles used non-linear models for growth curve analysis such as Brody, Richards, Von Bertalanffy, Gompertz and Logistic models were included. Studies that deal with the growth curve of goats, non-linear models for the growth curve of goats, and any articles that deal with the growth patterns of goats were included in the systematic review.

### Exclusion criteria

Articles were excluded if the requirements were not met such as, duplicate records, they studied other species, used other methods of growth analysis like linear models, and articles were not the full text but only the abstract.

### Data extraction

Ledimo Faith Makgopa and Thobela Louis Tyasi were independently extracted the data of the current study and reached a general agreement regarding all the materials. The articles that met the criteria had: author, year of publication, and type of model.

### Ethical considerations

Plagiarism, misconduct, informed consent, and data manipulation were considered ethical issues by all authors when performing this systematic review.

## Results

### Searched results

**Fig. 1:**
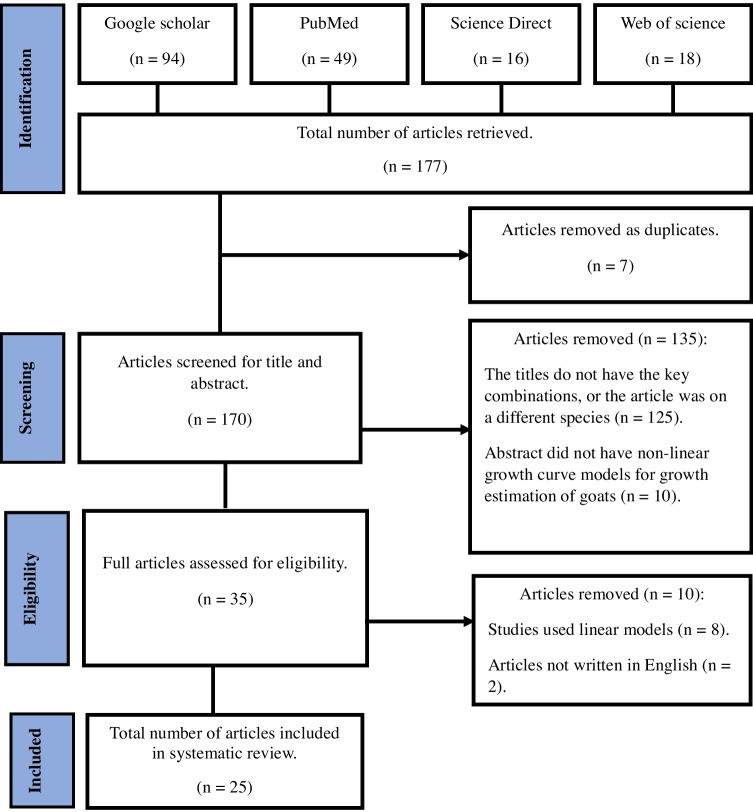
Flowchart of identification and selection of studies for systematic review

Figure 1 represents the flowchart of the identification and selection of studies for systematic review. In the primary search, a total of 177 articles were retrieved. After excluding 7 duplicate publications, 170 articles remained. The articles were screened for title and abstract, 135 articles were removed since the articles did not have the key combinations and the abstract did not have non-linear growth curve models for growth estimation of goats. About 35 articles were selected for full-text search and eligibility verification, and a total of 25 articles were included in this systematic review. The reason for exclusion of articles is stated in Fig. 1.

### Characteristics of included studies

A total of 25 articles were accessed and selected as meeting the criteria for inclusion in the review as indicated in Table 1. The results indicated that Das et al. (2016) and Paul et al. (2016) used the same number of goats (*n* =142) for their studies, in both studies the breed of the goats was not disclosed. The results showed that majority of the goat breed used in the 25 articles were indigenous goat breeds (*n* = 23). About 3 articles out of the 25 articles used goat breeds that produce mohair (Özdemir and Dellal 2009; Cak et al. 2017; Kheirabadi and Rashidi 2019) and one goat breed that produced cashmere (Ghiasi et al. 2018). The findings showed that Özdemir and Dellal (2009) and Cak et al. (2017) studied the growth patterns of the same goat breed the Angora goat breed. The results discovered that Lestari et al. (2020) and Sutopo et al. (2021) studied the growth patterns of the same goat breed (Kejobong).

**Table 1:** Characteristics of included studies

Authors	Years	Country	Goat Breed	Model	Environmental Condition
Abdelsattar et al	2021	China	Laiwu Black goat	Gompertz, Logistic, Quadratic model	Tropical
Araujo et al	2020	Brazil	Saanen goat and Anglo Nubian goat	Logistic	Tropical
Arre et al	2019	Brazil	Anglo-Nubian goat	Logistic and Brody	Tropical
Cak et al	2017	Turkey	Coloured Mohair goat	Gompertz and Richards	Mediterranean
Celik et al	2018	Pakistan	Daira Din Panah goat	Janoscheck	Temperate
Das et al	2016	India	-	Logistic	Tropical
de Sousa et al	2021	Brazil	Canindé goats	Brody	Tropical
Gaddour and Najari	2008	Tunisia	Damascus, Alpine, Indigenous, crosses goats	Gompertz	Mediterranean
Gautam et al	2019	Iran	Sirohi goat	Brody	Semi-arid
Ghiasi et al	2018	Iran	Raeini Cashmere goats	Gompertz	Semi-arid
Khan and Khatun	2013	Bangladesh	Black Bengal goaffts	Gompertz	Tropical
Kheirabadi and Rashidi	2019	Iran	Markhoz goat	Brody	Semi-arid
Lestari et al	2020	Indonesia	Kejobong goats	Von Bertalanffy	Tropical
Pires et al	2017	Brazil	Repartida goat	Logistic	Tropical
Magotra et al	2021	India	Beetal goat	Brody	Tropical
Najari et al	2010	Tunisia	Indigenous	Gompertz	Mediterranean
Ozdemir and Dellal	2009	Turkey	Angora goats	Logistic and Gompertz	Mediterranean
Paul et al	2016	India	-	Von Bertalanffy	Tropical
Rashad et al	2022	Egypt	Egyptian Damascus goats	Quadratic model	Tropical
Sutopo et al	2021	Indonesia	Kajobong goat	Von Bertalanffy and Brody	Tropical
Trach and Phiovankham	2011	Vietnam	Indigenous and crossbred goats	Gompertz	Tropical
Tyasi et al	2022	South Africa	Non-descript indigenous goats	Logistic 4P and Logistic 5P	Tropical
Waheed et al	2011	Germany	Beetal goats	Gompertz	Temperate
Waiz et al	2019	India	Sirohi goats	Brody	Tropical
Wiradarya et al	2020	Indonesia	Kacang goats	Gompertz	Tropical

-Not indicated

### Publications by year

**Fig. 2:**
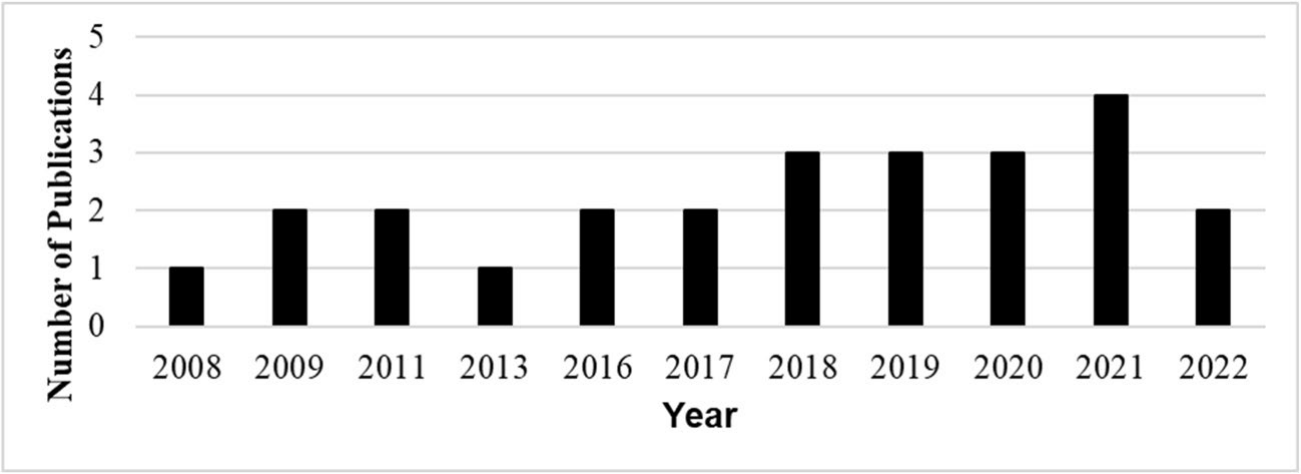
Publications by year

The results showed that out of 25 articles, the year 2021 had many numbers of articles published (*n* = 4) as shown in Fig. 2. The results also showed that the years 2008 (Gaddour and Najari 2008) and 2013 (Khan and Khatun 2013) had the least number of articles (*n* = 1).

### Publications by country

**Fig. 3:**
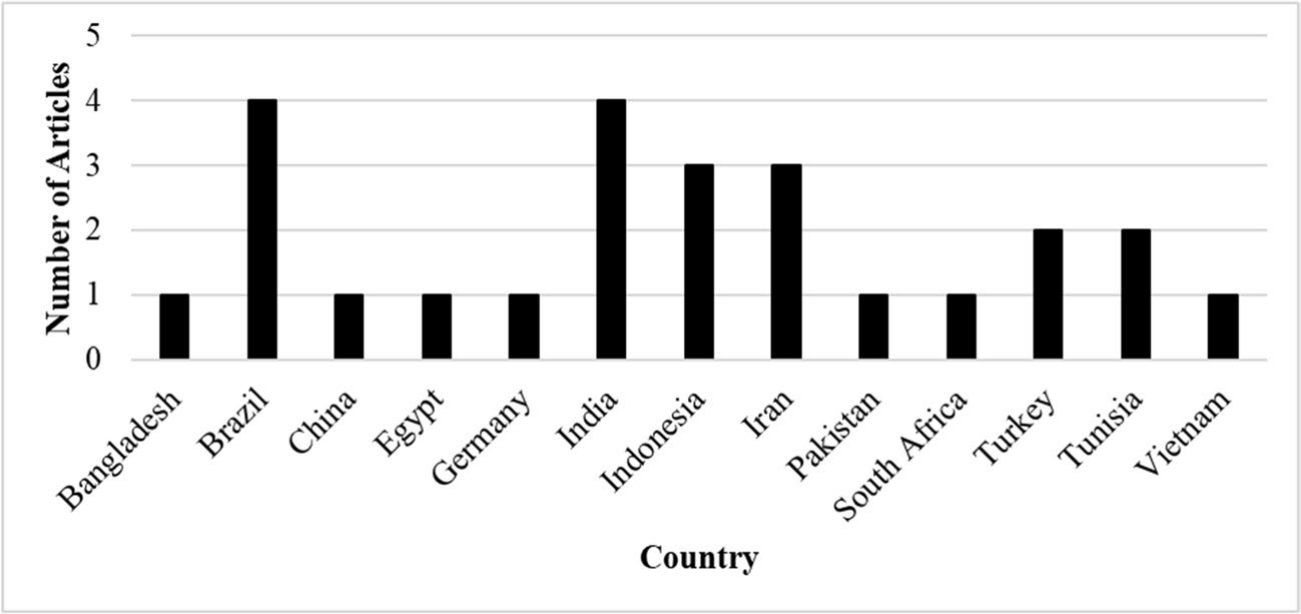
Publications by country

Figure 3 present the publications by country for the articles used in this review. The results indicated that out of 25 articles Brazil (Pires et al. 2017; Arré et al. 2019; Araújo et al. 2020; de Sousa et al. 2021) and India (Das et al. 2016; Paul et al. 2016; Magotra et al. 2021; Waiz et al. 2019) had the high number of articles (*n* = 4). The findings also showed that Germany (Waheed et al. 2011), Vietnam (Trach and Phiovankham 2011), Bangladesh (Khan and Khatun 2013), Pakistan (Celik et al. 2018), China (Abdelsattar et al. 2021), Egypt (Rashad et al. 2022), and South Africa (Tyasi et al. 2022) had the least number of articles (*n* = 1).

### Publications by environmental conditions

**Fig. 4:**
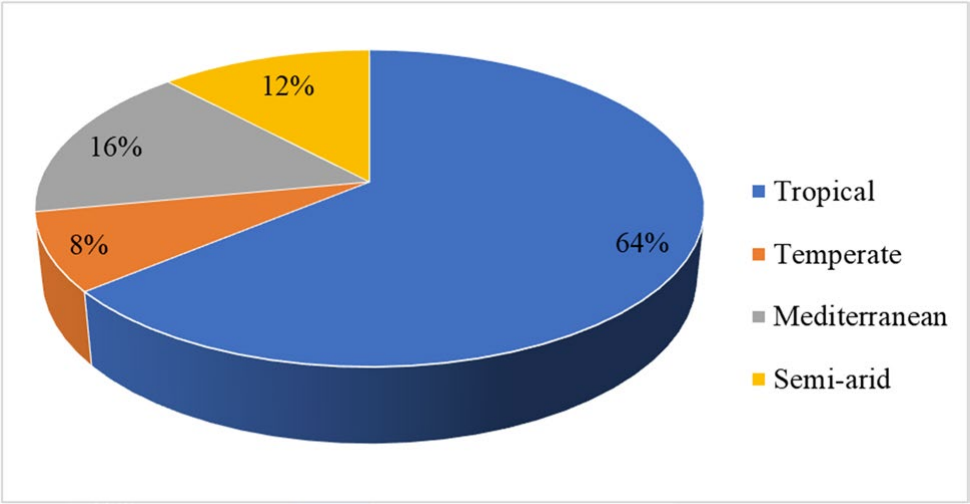
Publications by environmental conditions

Publications by environmental conditions are represented in Fig. 4. The results indicated that out of the 25 included articles, 64% (16/25) were studied in the tropical environment, 16% (4/25) were in the mediterranean environment. The findings further indicated that 12% (3/25) of the articles were studied in semi-arid environmental conditions (Ghiasi et al. 2018; Kheirabadi and Rashidi 2019; Gautam et al. 2019), while only 8% (2/25) of the articles were studied in the temperate environmental conditions (Waheed et al. 2011; Celik et al. 2018).

### Publications by growth curve models

**Fig. 5:**
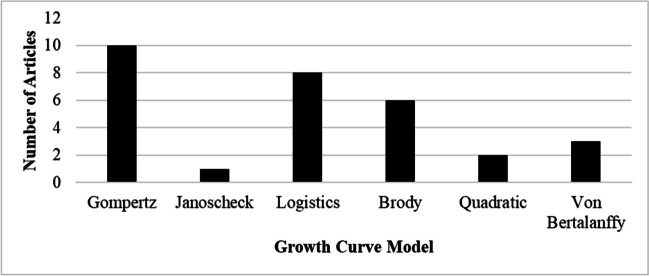
Publications by growth curve models

Publications by growth curve models are presented in Fig. 5. The results indicated that out of 25 articles, Gompertz growth curve model was the most used (*n* = 10), followed by Logistic (*n* = 8), then Brody growth curve model (*n* = 6). The results also showed that Janoscheck growth curve model was the least used model (*n* = 1) for estimation of growth curve parameters in goats (Celik et al. 2018).

### Goodness of fit for growth curve models

Table 2 represents the best goodness of fit for growth curve models used in the in the reviewed articles. Out of 25 reviewed articles, only 14 reported on the goodness of fit criteria for the selection of the best non-linear growth curve models. Coefficient of determination (*R*^2^) and Adjusted coefficient of determination (Adj.*R*^2^) were used as selection criteria for the best growth curve model. Out of 14 articles reported on the best model, only 11 articles used *R*^2^ for the selection of the best model, while 3 articles used Adj.*R*^2^ (Magotra et al. 2021; de Sousa et al. 2021; Tyasi et al. 2022). Out of 11 articles that used *R*^2^ to select the best non-linear growth curve model, Gompertz was used by only 10 articles to determine the growth curve parameters, however only 3 articles selected Gompertz as the best non-linear growth curve model, while logistic was used by 9 articles, but only 4 articles reported it as the best model. As indicated by the results out of 11 articles that used R^2^ for the selection of the best non-linear model, Celik et al. (2018) selected Janoscheck to be the best non-linear model and Rashad et al. (2022) selected Quadratic as the best non-linear growth curve model. Out of articles 3 articles that used Adj.R^2^ to select the best non-linear growth curve model, 2 articles used Brody, Gompertz, Logistic, and Von Bertalanffy, and both articles reported Brody to be the best non-linear growth curve model, followed by Von Bertalanffy, Gompertz, then Logistic (Magotra et al. 2021; de Sousa et al. 2021). As shown by the results, Tyasi et al. (2022) was the only article that used 3P Logistic, 4P Logistics, 5P Logistic, 3P Gompertz and 4P Gompertz non-linear growth curve models and reported that 5P Logistic model was the best non-linear model, while 3P Logistics, 4P Logistic, 3P Gompertz, 4P Gompertz were equally second-best non-linear growth curve models.

**Table 2:** Goodness of fit criteria for growth curve models

Author and Year	Breed	Sex	Goodness of fit	Model						
				Gompertz	Brody	Logistic	Richards	Quadratic	Von Bertalanffy	Janoscheck
Arré et al. 2019	Anglo Nubian goat	Female	*R*^*2*^	0.98	0.98	0.97	N/A	N/A	0.98	N/A
Abdelsattar et al. 2021	Saanen	-	*R*^*2*^	0.982	N/A	0.992	N/A	N/A	N/A	N/A
	Anglo Nubian		*R*^*2*^	0.978	N/A	0.998	N/A	N/A	N/A	N/A
Cak et al. 2017	Singleton (Coloured Mohair goat)	Both	*R*^*2*^	99.70	N/A	N/A	99.70	N/A	N/A	N/A
	Twin (Coloured Mohair goat)	Both	*R*^*2*^	99.53	N/A	N/A	99.57	N/A	N/A	N/A
Celik et al. 2018	Daira Din Panah goat	-	*R*^*2*^	N/A	N/A	0.989	N/A	N/A	0.997	0.999
de Sousa et al. 2021	Caninde goats	Both	*Adj.R*^*2*^	0.790	0.794	0.785	N/A	N/A	0.792	N/A
Gautam et al. 2019	Sirohi goat	Male	*R*^*2*^	96.8	98.6	95.0	N/A	N/A	97.5	N/A
		Female	*R*^*2*^	97.2	98.8	95.3	N/A	N/A	97.8	N/A
Kheirabadi and Rashidi 2019	Markhoz goat	-	*R*^*2*^	0.971	0.975	0.964	N/A	N/A	0.973	N/A
Magotra et al. 2021	Beetal goat	Both	*Adj.R*^*2*^	0.992	0.999	0.975	N/A	N/A	0.996	N/A
Özdemir and Dellal 2009	Angora goat	-	*R*^*2*^	0.956	N/A	0.957	N/A	N/A	N/A	N/A
Pires et al. 2017	Repartida goat	Both	*R*^*2*^	0.9097	0.8753	0.9147	0.8988	N/A	0.8858	N/A
Rashad et al. 2022	Egyptian Damascus gost	-	*R*^*2*^	0.685	0.678	0.683	N/A	0.731	0.685	N/A
Tyasi et al. 2022	Non-descript indigenous goats	-	*Adj.R*^*2*^	3P-0.989 4P-0.989	N/A	3P-0.989 4P-0.989 5P-0.991	N/A	N/A	N/A	N/A
Waiz et al. 2019	Sirohi goat	Male	*R*^*2*^	97.7	99.1	96.1	97.7	N/A	N/A	N/A
		Female	*R*^*2*^	98.1	99.4	96.5	98.0	N/A	N/A	N/A
Wiradarya et al. 2020	Kacang goat		*R*^*2*^	0.98	N/A	0.98	N/A	N/A	N/A	N/A
		Female	*R*^*2*^	0.98	N/A	0.98	N/A	N/A	N/A	N/A

-, Not indicated; N/A, not studied; *R*^2^, coefficient of determination; Adj.*R*^2^, Adjusted coefficient of determination

## Discussion

Growth curve parameters are useful during the process of selection according to animal performance and culling of underperforming animals to achieve genetic progress (Arré et al. 2019). Growth curve parameters can also be used when farmers are examining the effect a certain treatment has on the growth performance of their breed (Pires et al. 2017). Understanding the growth curve parameters of goats at different production stages is important when farmers want to implement management strategies such as feeding, slaughter age and genetic improvement of the species to increase their production potential (Ghiasi et al. 2018). However, the best non-linear models for estimation of growth patterns are not yet known. The systematic review was conducted to reveal the best non-linear models used on estimation of growth patterns in goats in 25 included articles. The results showed that out of 25 articles, Gompertz growth curve model was the most used (10/25) followed by Logistic (8/25). However, Waheed et al. (2011) estimated growth curve parameters in Beetal goats applying Brody and Gompertz models and concluded that these growth curve models are equally the best non-linear models for describing the growth of Beetal goats. Gompertz model is a type of non-linear mathematical model for a time series which was named after Benjamin Gompertz (1779–1865) that is a sigmoid function which describes growth as being slowest at the start and end of a given period and it is commonly used in animals and plants (Ghiasi et al. 2018). This study revealed that the 40% of the articles recommended Gompertz model as the most suitable mathematical model for growth patterns of goats were originated in tropical environmental countries such as Vietnam (Trach and Phiovankham 2011), Bangladesh (Khan and Khatun 2013), Indonesia (Wiradarya et al. 2020) and China (Abdelsattar et al. 2021). Another 40% of articles were from mediterranean environmental countries such as Tunisia (Gaddour and Najari 2008; Najari et al. 2010), Turkey (Özdemir and Dellal 2009; Cak et al. 2017). While 10% of articles were from Germany as temperate environmental country (Waheed et al. 2011) and another 10% were from Iran as semi-arid environmental country (Ghiasi et al. 2018). To the authors’ knowledge, this is the first systematic review reporting on the estimation of growth patterns in goats using non-linear growth curve models. Hence, there is no comparison of other systematic review findings in the topic. The implication of this systematic review is that the Gompertz model can be used on estimation of growth patterns of goats in different countries with different environmental conditions. Strength of this review was that no similar study had been conducted on estimation of growth curve in goats. The contribution of this systematic review to the body of knowledge is to suggest Gompertz as a non-linear model for prediction of growth patterns in goats. This systematic review has some limitations that need to be mentioned: (1) A total of 6 articles from 25 included did not indicate the sex of the goats which make it difficult to draw the conclude for sexes. (2) No similar data was found to proceed to meta-analysis. Arré et al. (2019) compared different nonlinear models for describing the growth of Anglo Nubian does in Brazil and concluded that Gompertz was one of the best models. Therefore, Gompertz might be good for describing the growth of female goats. However, more studies need to be done on the to validate these findings. In conclusion, Gompertz model is the most suitable non-linear growth model followed by Logistic for the prediction of the growth patterns in goats. Therefore, researchers should include the Gompertz and Logistic models when predicting the growth parameters of the goats.

## Declarations

### Additional information

No additional information is available for this manuscript.
